# Impact of the De-Alloying Kinetics and Alloy Microstructure on the Final Morphology of De-Alloyed Meso-Porous Metal Films

**DOI:** 10.3390/nano4040856

**Published:** 2014-10-17

**Authors:** Bao Lin, Lingxue Kong, Peter D. Hodgson, Ludovic F. Dumée

**Affiliations:** Institute for Frontier Materials, Deakin University, Waurn Ponds, VIC 3216, Australia; E-Mails: lingxue.kong@deakin.edu.au (L.K.); peter.hodgson@deakin.edu.au (P.D.H.); ludovic.dumee@deakin.edu.au (L.F.D.)

**Keywords:** de-alloying (DA) kinetics, through pores formation, metal surface texture, micro-structure morphology relationship

## Abstract

Nano-textured porous metal materials present unique surface properties due to their enhanced surface energy with potential applications in sensing, molecular separation and catalysis. In this paper, commercial alloy foils, including brass (Cu_85_Zn_15_ and Cu_70_Zn_30_) and white gold (Au_50_Ag_50_) foils have been chemically de-alloyed to form nano-porous thin films. The impact of the initial alloy micro-structure and number of phases, as well as chemical de-alloying (DA) parameters, including etchant concentration, time and solution temperature on the final nano-porous thin film morphology and properties were investigated by electron microscopy (EM). Furthermore, the penetration depth of the pores across the alloys were evaluated through the preparation of cross sections by focus ion beam (FIB) milling. It is demonstrated that ordered pores ranging between 100 nm and 600 nm in diameter and 2–5 μm in depth can be successfully formed for the range of materials tested. The microstructure of the foils were obtained by electron back-scattered diffraction (EBSD) and linked to development of pits across the material thickness and surface during DA. The role of selective etching of both noble and sacrificial metal phases of the alloy were discussed in light of the competitive surface etching across the range of microstructures and materials tested.

## 1. Introduction

Meso-porous metal frameworks offer enhanced specific area, surface energy, thermo-chemical stabilities and electron transport compared to bulk metals due to surface vacancies formed on the nano-textured metal surface [1]. These frameworks were demonstrated to be promising materials with potential application as electro-chemically or chemically driven actuators [2,3,4], electrodes [5,6,7], catalytic reactors [8,9,10,11], heat exchangers [12], as reinforcement skeletons across composite materials, but also for their biocompatibility as biomedical prosthesis and as membranes for liquid purification [13].

Macro-porous metal materials, with through pores ranging from the tens to hundreds of micrometers [14], can be fabricated on industrial scale by molten metal foaming or metal particle sintering, two highly mature technologies [14,15,16]. The electro-chemical and thermo-mechanical stabilities of such porous metal based materials were shown to be superior to that of most porous ceramic and polymeric materials when operated in organic solvents, concentrated salt solutions and high temperature [17,18], thus allowing for their sterilization or operation in steam or high temperature environment. The formation of sub-micron through pores across these porous metal materials is however compromised due to physical fabrication constraints preventing their application at the nano-scale level [19]. The design of long range meso-pores by foaming and sintering is challenging due to the respective segregation foaming agent [1] and limitations related to particle size distributions and surface coalescence mechanisms for both foaming and sintering, respectively [1,20], which may lead to poorly interconnected pore networks or to a large degree of densification of the metal matrix [1]. These issues are hindering the development of open, continuous and highly porous meso-porous metal structures for industrial applications requiring the development of novel and more refined fabrication techniques. Recently, the development of a range of novel fabrication techniques [21] including electroless deposition [20,22,23,24], metal nano-particle self-assembly sacrificial dense matrixes [25,26,27,28], electro-spinning [1], nano-foaming and layer-by-layer self-assembly [29] were shown to lead to the formation of highly interesting structures. Although promising, these techniques have a typically low through-put and still offer limited scope for the processing of large surface area of meso-porous metal materials.

Chemical de-alloying (DA) of metal alloys was shown to lead to the formation of surface pores within the range of 3–5 μm [30], and also recently used to form meso-porous metal nano-particles hierarchical [13,31] or core-shell meso-porous metal structure [32]. DA is a top-down materials synthesis approach where no substrate is required thus allowing for a highly versatile process [1]. As opposed to the structures obtained from foaming or sintering, de-alloyed porous structure offer much finer pore size distribution, potentially larger porosity and an highly interconnected pore network with lower porosity [33]. The pore size distribution, porosity and pore interconnectivity obtained across the metal materials may be tuned by varying the material composition, properties and the etching conditions [34,35]. The alloy composition, the number of phases and the material micro-structure will greatly affect the dynamics of the DA process, pitting initiation on the metal surface, and pore propagation [36]. Furthermore, processing parameters such as the etchant corrosivity, concentration, and exposure time of the metal sample in solution will also affect the kinetics of DA and therefore the solvation and reorganization of locally displaced or etched atoms from the grains across the surface or pores of the alloy [37]. The microstructure of the alloy material, and thus both metal grain size distribution and localized composition will highly rely on the material fabrication process, such as electron beam evaporation [38], electroplating [39], physical vapor deposition [31], industrial rolling [40], melt-spinning [41,42,43,44,45], and on potential post-treatments, such as rolling or annealing. The history of the material and thus the isotropy of the grains orientation across the thickness and surface of the material will affect the final morphology and pore formation across the materials.

Pure metals are typically considered noble if their standard electrical potential *E*^0^, *i.e.*, their first level of oxidation, is lower than 0 V corresponding to the potential of hydrogen [46]. A suitable precursor alloy should be made of a noble metal which will compose the framework material of the de-alloyed porous structure [47]. DA of an ideal, atomically smooth, and highly crystalline surface will therefore start from localized surface pitting across the metal grains richer in the less noble atoms. Pitting will enhance the specific surface area of the material and generate vacancies [48] across the surface which will propagate through the thickness of the material, creating islands of noble metal [37]. The average distance between two noble islands, called the critical spacing λ, will not dramatically change during the process across grains made of a homogenous single phase material exhibiting a continuous microstructure [49]. The second stage of the DA process will be the coarsening of the islands within the material pores by localized coalescence by stripping surface atoms present across the proto-pores forming deeper within the material. In the third stage of the DA process, only the less noble atoms are being dissolved and removed from the surface of the pits. The critical spacing between two agglomerates will then increase leading to the formation of distinct surface ligaments progressively joining and extending into a continuous nano- or meso-porous network made of solely the noble metal phase [50,51,52].

Metal alloys such as gold (Au), silver (Ag) and palladium (Pd) based were successfully used for the formation of purely nano- or meso-porous materials [40,45]. The cost of these materials, however, is prohibitive towards the mass fabrication of metal membranes and cheaper materials should be investigated. Although copper (Cu) based alloys of interest due to their lower cost and high thermal conductivity were also successfully converted, the formation of highly ordered meso-porous Cu based materials is rendered more difficult than for more noble metals due copper oxide formation on the material surface stopping charge transfers across electronic vacancies generated upon sacrificial metal atoms solubilization [46,53,54]. Despite these promising perspectives, the formation of meso-porous thin films depth across these materials has not been extensively studied to date and most published works focused on sub-micron thick alloy films. The design of meso-porous materials with through pores is therefore a challenge to be overcome in order to process long range order pores and large surface area materials for industrial scale applicability.

In this paper, meso-porous Cu and Au materials were processed by chemical DA of pristine Cu-Zn and Au-Ag alloy thin films. The impact of different DA conditions, such as etching pH, exposure time, and temperature on the final material morphology and on the residual least noble metal constituent were for the first time systematically investigated as a function of the thickness of de-alloyed thin films to evaluate the consistence of the pore formation process. The impact of the alloy microstructure, grain distribution and grain boundaries on the final de-alloyed material morphology were also analyzed by crystallography analysis with electron back-scattered diffraction (EBSD) by focus ion beam (FIB) surface patterning the surface of the materials prior to performing DA across the series of samples. Furthermore, the impact of metal ion solvatation in solution and the formation of a concentration gradient across the forming meso-pores and of metal oxide particles on the surface of the material as well as the relationship between pore sizes, pore penetration depth and the material microstructure variations of pristine membrane will be critically discussed.

## 2. Results and Discussion

### 2.1. Kinetics of the De-Alloying Process and Structure-Morphology Relationship

**Figure 1:**
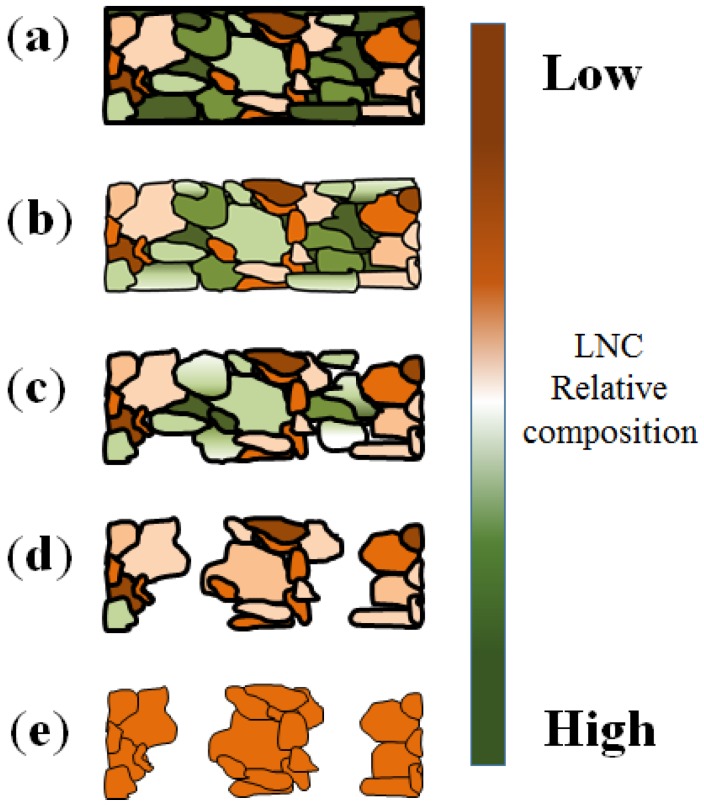
Schematic of the de-alloying (DA) process and pore propagation from the as a function of the grain distribution and composition: (**a**) pristine alloy microstructure with two alloy constituents distributed and mixed across a number of grains; (**b**) surface pitting and pore initiation; (**c**) DA of less noble atom rich exposed grains; (**d**) pore progression and through pore formation; and (**e**) final nano-porous materials. LNC: less noble content.

As shown in Figure 1, the DA process consist of four distinct steps. First, surface pitting is initialized on the surface of the alloy. It is typically assumed that pitting occurs preferentially across the lower crystallinity points corresponding to grain boundaries [55]. Then, the less noble metals constituting the alloy are selectively etched from the surface of the pitting points. The relative composition, *i.e.*, the amount of the most noble metals across the grains will condition pore propagation and pore wall formation [34,45,56]. Therefore pits starting at the interface between less concentrated and richer grains in noble metal will develop into pores and form pure metal walls, typically called ligaments, while the growth of pits started across more noble rich grain may be prematurely stopped. The third step in the DA process consist in the development of pores from the surface of the material. The dissolution of the least noble atoms will lead to the formation of pathways within atomic re-arrangement of noble atoms. Dissolved species will be flushed away into the carrier solvent, typically water. Theoretically, the process should continue until the complete removal of the least noble metal atoms thus forming, if the relative content of less noble metal atoms is high enough, through pores across the material thickness. A relatively limited content of less noble metal atoms may remain encapsulated across the crystalline structure of the alloy if present in small concentrations within high concentration noble atoms ligaments.

**Figure 2:**
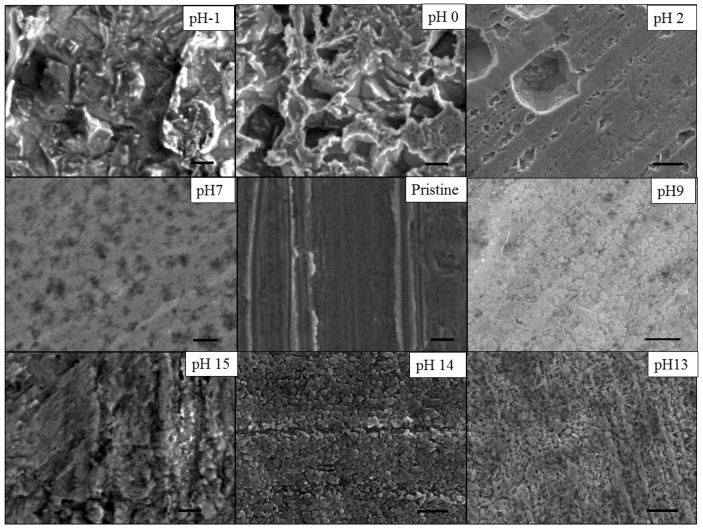
The scanning electron micrograph (SEM) images (in-plane view) of de-alloyed samples which de-alloyed with different etching solution (cross-section view has shown in Figures S2 and S3). 10 M HCl (pH = −1), 1 M HCl (pH = 0), 0.01 M HCl (pH = 2), deionized (DI) water (pH = 7), 1 × 10^−5^ M NaOH (pH = 9), 10 M NaOH (pH~14), 1 M NaOH (pH = 14) and 0.1 M NaOH (pH = 13). Scale bars all correspond to 500 nm.

The impact of the DA process parameters on the pore morphology was also systematically investigated to evaluate the optimal DA conditions to form narrow distribution and through pores across the alloyed material. The impact of the solution pH on the selective etching and surface pitting across CuZn_30_ thick metal alloy films is shown in Figure 2. The *E*^0^ potential of Cu/Cu^+^, Cu/Cu^2+^ and Zn/Zn^2+^ are +0.340 V, +0.520 V and −0.7628 V, respectively, while the pKd corresponding to the dissolution pH or redox potential of the Cu/CuO and Zn/ZnO species are typically comprised between pH 9 and 13 and pH 7 and 15 respectively depending on the type of etchant and solvation media (Figure S1). Cu would however be etched by highly oxidizing acids [57], such as nitric acid, sulfuric acid and hydrochloric acid [58] and pH alone is therefore not sufficient to evaluate competitive etching. In our case, it is expected that selective DA of the Zn from the CuZn single phase material should occur over a pH range between 13 and 15. Previous work published on the DA of Cu-Zn alloys were however performed in HCl or NaOH solutions at pH of below 1 or above 13 [54,59,60] which was possible due to differences in etching rate between Cu and Zn in nearly pure HCl.

As shown on the scanning electron micrographs (SEMs) in Figure 2, the value of the pH is found to highly affect the etching selectivity which dramatically affects the surface morphology of the metal thin films.

**Figure 3:**
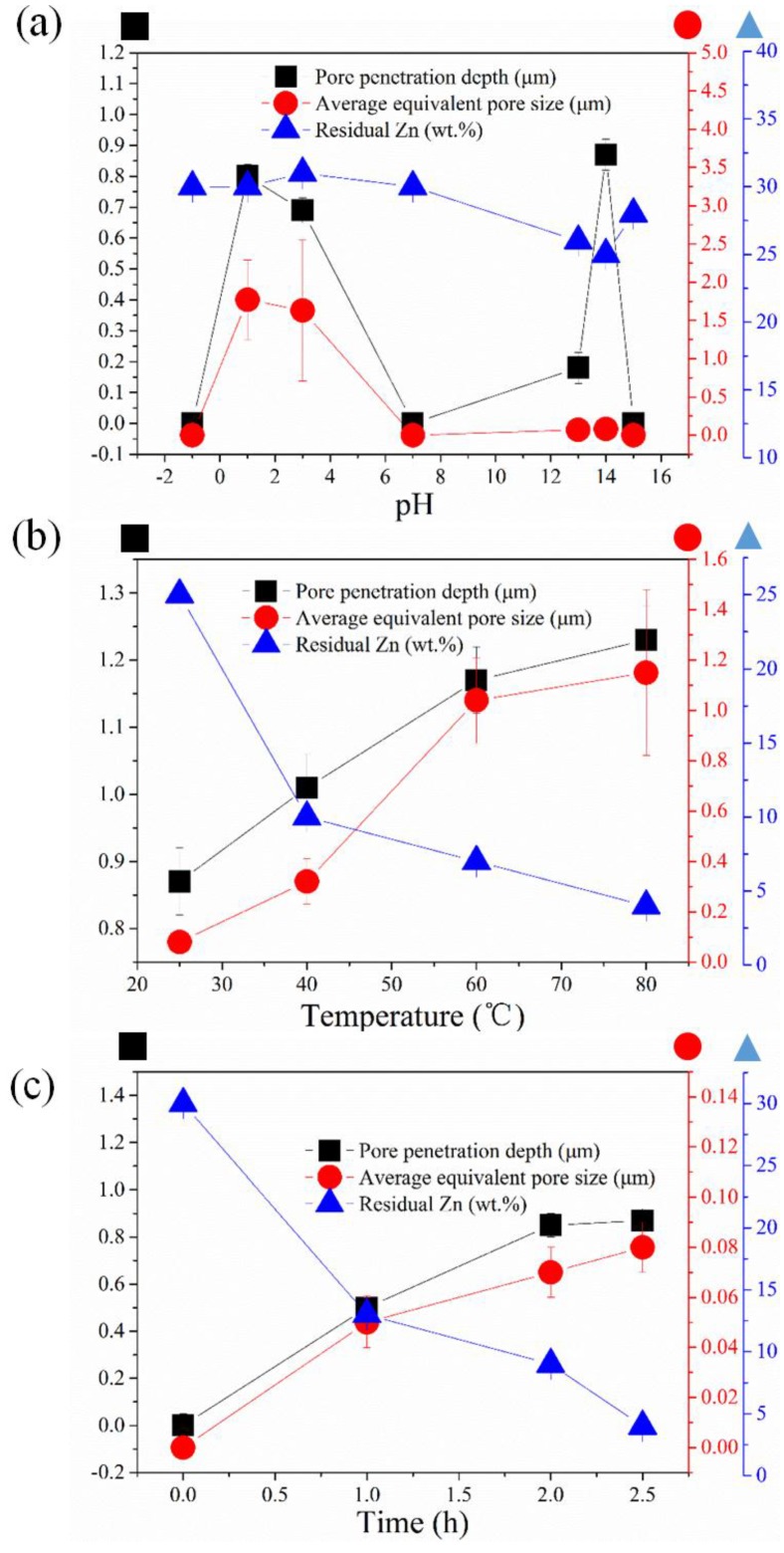
The impact of DA condition on surface pore size, penetrate depth and residual Zn content from energy dispersive spectroscopy (EDS): (**a**) pH value-based series de-alloyed foil de-alloyed with different etching solutions (10 M HCl (pH = −1), 1 M HCl (pH = 1), 0.01 M HCl (pH = 3), 10 M NaOH (pH = 15), 1 M NaOH (pH = 14) and 0.1 M NaOH (pH = 13)) for 2.5 h at 25 °C; (**b**) temperature-based series de-alloyed foil de-alloyed with 1 M NaOH for 2.5 h; and (**c**) time-based series de-alloyed foil de-alloyed with 1 M NaOH at 60 °C.

As seen in Figure 3a, the pore size distribution is quite large upon using an acidic etching solution.

At extreme pH below 0 or beyond 14, where water is not anymore the main solvent, DA is found to be non-selective and both Cu and Zn are etched away from the alloy. The morphology of the sample surface at pH < 1 is very rough with clear evidence of surface corrosion and neither obvious ligament nor long range patterns formation. Although non-selective, the acidic etching process lead to deep and large distribution pores with an average pore size estimated from the SEMs at ~1 µm (Figure 2). At pH = 7, the sample is shown to be largely unaffected by water and no obvious trace of corrosion of pitting could be seen. However at alkaline pH values between 13 and 14, the surface is found to progressively be composed of nodules which were shown by energy dispersive spectroscopy (EDS) (Figure S4) to be primarily composed of Cu. The size of the spaces between these nodules, and thus the surface pore size and porosity, are found to also increase with pH. However, pH above 14, the DA process is again found to be non-selective and to lead to a more randomly textured surface. The pore size distribution for the alkaline DA process was also found to be narrower than that obtained for the acidic treatment which is a more preferential rearrangement for the recombining of Cu atoms and Cu ions at these concentrations (Figures S2 and S3).

**Figure 4:**
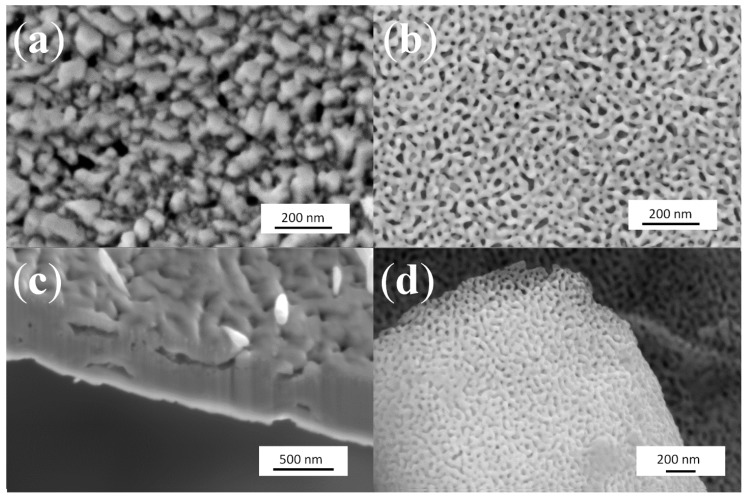
SEM image of de-alloyed CuZn_15_ and AuAg_50_: (**a**) DA CuZn_15_ with 1 M NaOH for 1 h at room temperature; (**b**) DA AuAg_50_ with 70% Nitric Acid for 10 min at room temperature; (**c**) cross-section view of (**a**); and (**d**) cross-section view of (**b**).

Interestingly, this phenomenon is also found to occur for the CuZn_15_ ultra-thin foils. The formation of the pores across the foils, which were only 150 nm thick, was also found to generate nodules linked by large and thick ligaments (Figure 4a,c). Similar experiments performed on the AuAg_50_ leaves with selective etching at 70 wt% nitric acid showed on the other hand the formation of highly ordered ligaments and interconnected pore networks (Figure 4b,d).

**Figure 5:**
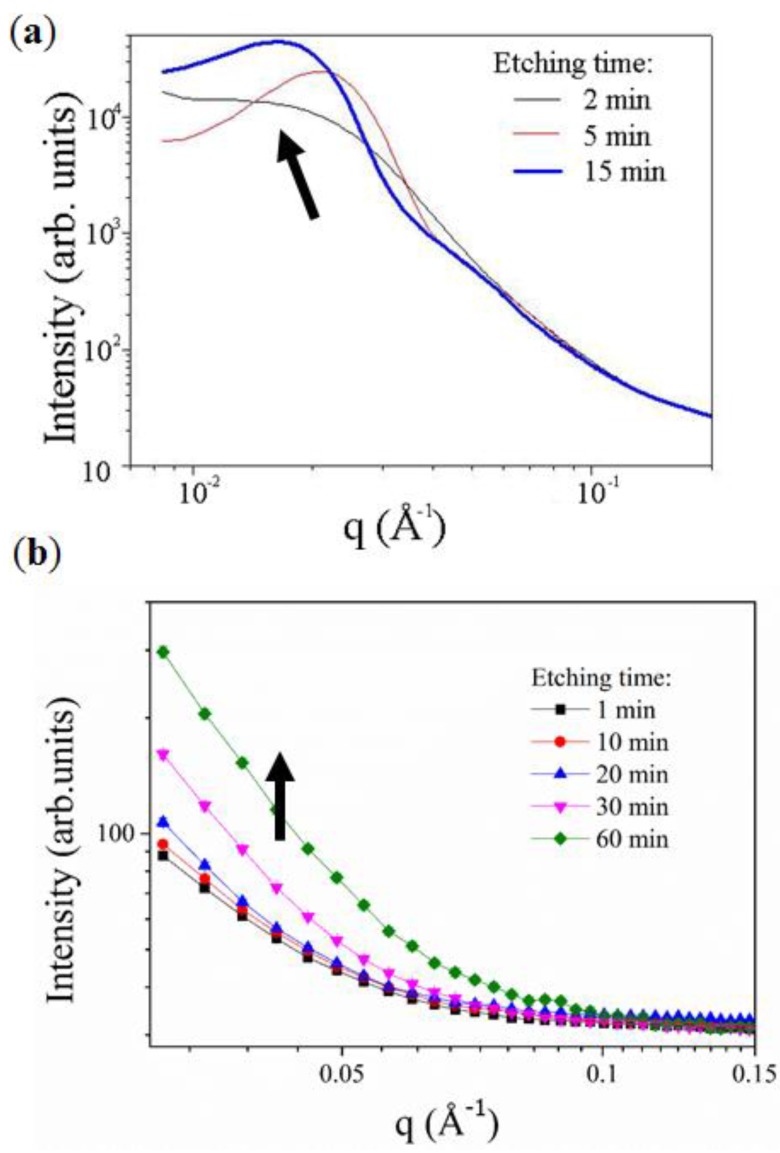
Small angle X-ray scattering (SAXS) analysis of *in situ* DA experiment on: (**a**) AuAg_50_; and (**b**) CuZn_15_.

The evaluation of the ligament distribution was performed by small angle X-ray scattering (SAXS) analysis (Figure 5). It can be clearly seen that the ligament distribution across the CuZn_15_ although evolving as a function of the etching time is extremely broad (17–64 nm) while that of the AuAg_50_ is narrow and stabilizing at approximately 30 ± 2 nm. This result supports previously reported data on SAXS of similar AuAg metal ultra-thin films [52] where the ligaments were shown to coarsen with increasing DA time. Furthermore, the specific surface area of the CuZn_15_ samples are shown to increase from 0.0075 ± 0.0037 m^2^/g before DA to 0.7753 ± 0.0840 m^2^/g after DA. This represents more than 200 times of increase and highlights the change of roughness and the formation of a porous structure. The specific surface area of the AuAg_50_ increased from 1.6017 ± 0.2566 m^2^/g to 30.3821 ± 3.324 m^2^/g after DA. This represents an 18 times increase and demonstrate the formation of the ligaments across the material. The metal atom distribution after DA, where both metal oxides and pure metal co-exist on the material surface may explain the discrepancies between the materials. Indeed, although for the de-alloyed Au-Ag samples the surface is composed exclusively of Au, the alloy microstructure and the formation of copper oxides and the potential segregation of zinc across the Cu-Zn 15 may lead to localized variations of surface energy and thus of N_2_ adsorption capabilities. This may explain the large standard deviation for the later samples.

**Figure 6:**
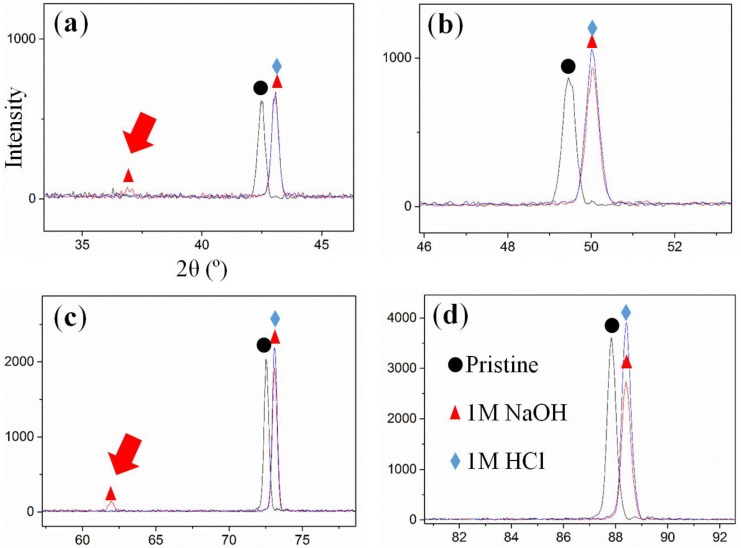
X-ray diffraction (XRD) patterns of de-alloyed CuZn_30_ foil de-alloyed with 1 M NaOH and 1 M HCl: (**a**) 2θ = 35°–45°; (**b**) 2θ = 46°–53°; (**c**) 2θ = 62°–77°; and (**d**) 2θ = 81°–92°.

X-ray diffraction (XRD) patterns for the CuZn_30_ before and after DA are presented in Figure 6. Although the characteristic peak of CuZn_30_ at 42.47°, 49.45°, 72.51° and 87.83° is shown to shift to 43.05°, 50.03°, 73.09° and 88.41° upon DA, it does not overlap with that of pure Cu [61]. This indicates that although partially dissolved from the material, Zn was not completely removed and that Cu atoms were rearranged during the DA process. Two new peaks are also found to appear upon DA in NaOH which are characteristic to CuO/Cu(OH)_2_ which may be generated on the surface from dissolved metal ions oxidized in air upon drying after the DA process or as shown in Figure S1 due to the passivation of Cu at these high pH in solution. Oxides were also detected in SAXS during the DA of the CuZn_15_ (Figure S5). The pore size distributions of these materials evaluated by SEM analysis suggest that HCl is not a suitable etching agent and that the pH of the solution should be carefully controlled to prevent non-selective etching. Furthermore, large quantities of Cu oxides and hydrates were formed in these acidic conditions which would ultimately affect the materials surface properties and reduce thermo-electrical properties and surface roughness. As seen in Figures S6 and S7, the thermal conductivity of the samples was shown to strongly reduce between 75 W·m^−1^·K^−1^ and 38 W·m^−1^·K^−1^ due to poor contact of rough surface. It is, therefore, expected that high pH is preferential for the DA of CuZn alloys.

The impact of the solution temperature on the DA kinetics was also assessed. It is found that the final pore size distribution is always ultimately ranging statistically around 1 μm regardless of the solution temperature between 25 °C and 80 °C. An increase of the solution temperature however led to a larger pore penetration depth with an initial 50% increase between 25 °C and 60 °C prior to plateauing above 60 °C up to 80 °C (Figure 3b). The plateauing of the pore penetration at higher temperatures may be due to the faster solubilization of Cu thus leading to a progressive but yet steady reduction of the alloyed material thickness. This hypothesis was found to be largely confirmed by the cross-sections SEMs (Figure S8) where the thickness of the porous materials was found to be reduced by nearly 2.4% between 60 °C and 80 °C. At higher temperature the etching of Zn is therefore less selective and the material is homogeneously corroded in a similar way as that depicted for low pH DA.

It is clear from the SEMs shown in Figure 3b,c, Figure S2 and Figure S3 that the pore penetration as a function of the DA exposure time or pH is largely plateauing around 1.2–1.5 µm (Figure S9). This is interesting and suggests that either the formation of a thin Cu oxide layer on the surface of the pores is preventing DA or that the concentration of the Zn metal ions within the pores, increasing within the initial moments of the pore propagation is slowing down thermodynamically the process. The increase of the Zn ion concentration would translate into an important change of the Zn activity in the solution which could indeed reduce the driving force of the solubilization. The residual Zn content is indeed found to plateau regardless of the etching time, pH or solution temperature of solution or prolonging the etching time. It is, however, worth noting that through this EDS only a surface down to approximately 1–2 µm may be probed and that simultaneously the roughness of the DA surface is strongly reducing the elemental count rate due to multiple angles secondary electrons scattering from the material surface. This is thus limiting the scope of the EDS analysis. The EDS analysis of low pH DA CuZn_30_ materials however offered consistent composition to that obtained on the reference pristine alloy suggesting that the orbital distribution obtained by EDS is nevertheless reasonably accurate at this micron-scale (Figure 6a). Therefore and due to the low surface Zn content plateauing around 5 wt% after 150 min, the Zn wt% evaluated from the EDS is likely lower than the actual total content. This hypothesis is also further confirmed by the XRD results previously shown suggesting that the peak shifts were not fully overlapping that of pure Cu.

### 2.2. Maximum Pore Penetration and Material Thickness Change during De-Alloying

**Figure 7:**
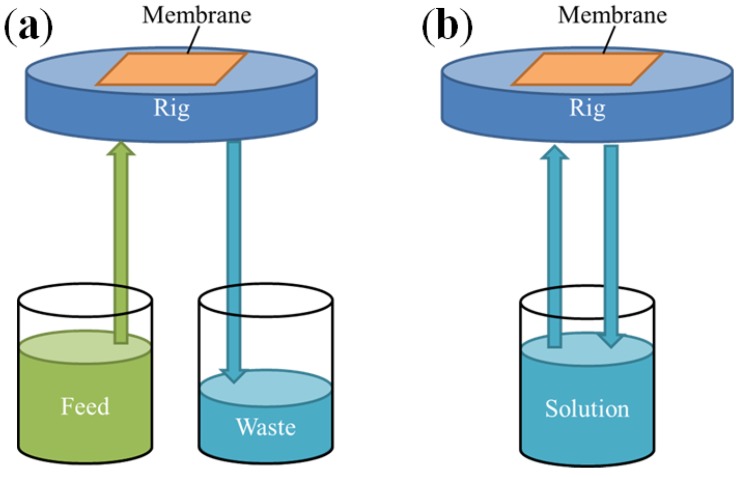
Schematic of the cross-flow contact rig: (**a**) non-recirculated flow; and (**b**) reference recirculated solution configurations.

In order to assess the true impact of the concentration polarization (CP) of the metal ions and etchants on the pore penetration depth, DA tests were performed with a cross-flow cell (Figure 7). The metal ions concentrations across the pores was thus carefully controlled and reduced to a minimum by flushing away de-alloyed ions. Although the CP within inside the pores cannot be fully avoided due to permanent solubilization of the Zn ions and geometrical constraints within such confined volumes, the kinetics of the selective etching should be affected if Zn ions were permanently and readily removed through high shear etching solution flow rate on the surface of the material. The etching rate of Zn will be faster than that of Cu within the right pH range which should therefore provide a platform for selective etching.

**Figure 8:**
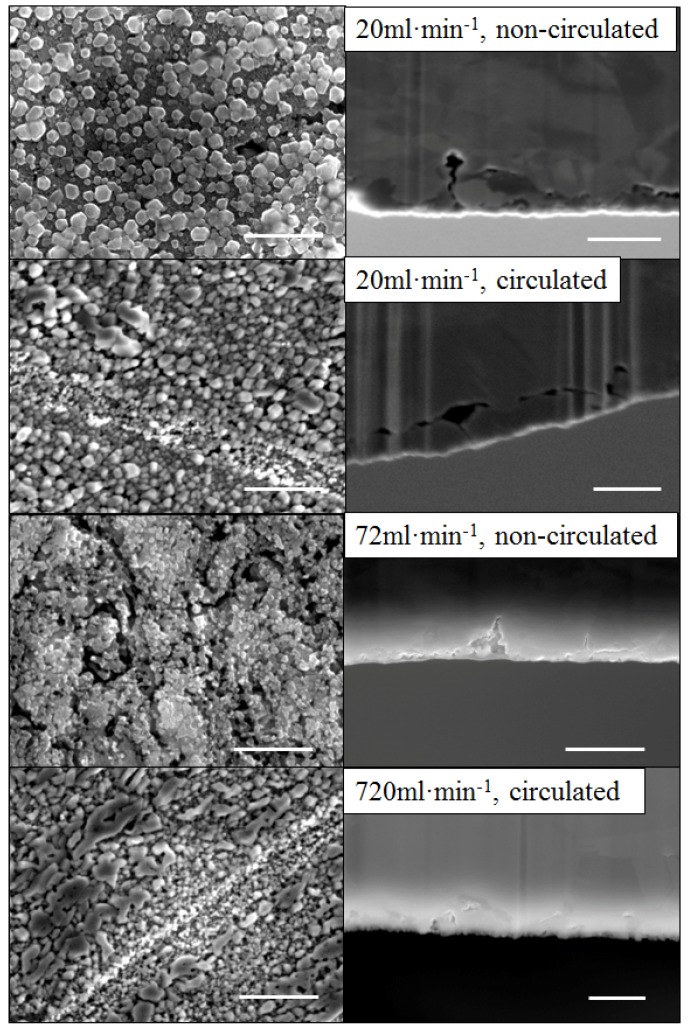
Surface and cross-section views of 1 M NaOH de-alloyed thin films with respective cross-flow surface velocities. Scale bars for the surface and cross section views correspond to 500 nm and 10 μm, respectively.

In the first series of tests the etching solution was therefore not recirculated after contacting the alloy thin film to prevent contamination and fresh solution permanently brought in contact with the alloy thin film. As seen in Figure 8, a sharp increase of the pore penetration was observed at 25 °C for the samples DA in this system where the solution was not recirculated. The reduction of CP by cross-flow rig led to a nearly 50% pore penetration increase with a reference dipping test from 0.87 μm to 1.25 μm. Benchmark tests were performed by recirculating the solution in order to assess the impact of solely surface shear flow on the pore penetration. As seen in Figure 7, the penetration depth for this tests were statistically similar to the dipping tests clearly highlighting the benefit of the on-recirculation. However, at high surface shear rate, namely 72 mL·min^−1^, the surface of the sample was found to crack. This was not attributed directly to the shear flow but to the fact that Cu ions were not provided sufficient time to re-arrange upon Zn leaching out from the material. The Cu were thus directly flushed from the surface limiting the efficiency of the DA process. This result was confirmed by the blue color of the solution building up over time in this configuration suggesting that the concentration of Cu ions was steadily increasing (not shown here). The surface shear flow also presented another advantage to the dipping tests. As seen in Figure 7, the low flow rate shear flow, while not mechanically compromising the films also limited the Cu oxide precipitation by preventing their formation. These tests infer that the DA process is highly sensitive for less noble metals than Au or Pd and that unlike previously reported studies the pore penetration depth is a critical parameter to investigate in order to assess properly the DA kinetics.

A mechanism depicting the DA process and through pore formation is proposed (Figure S9). In the case of CuZn_30_ thin film alloys DA is shown to largely lead to only surface texture and penetration depth to plateau at a fixed critical length found to largely rely on the activity of the Zn ions solubilized in solution (Figure S9a–c). Then, if the thickness of the thin film is small enough, *i.e.*, likely below the 5 µm benchmark for the composition presented here, through pores may be formed through side-to-side pore bridging. It is hypothesized that after through pore formation, the thickness and of the film should plateau while pore diameter may be coarsened due to pore surface etching (Figure S9c–e). This is suggested by the dynamics observed on the SAXS patterns presented in Figure 4 for both AuAg_50_ and CuZn_15_ ultra-thin films. Even after through pore formation, the pore size distribution is found to broaden until complete solubilization of the least noble phase, *i.e.*, Ag [52]. DA of CuZn thin films prepared with the same fabrication route and exhibiting various Zn contents would be highly interesting in order to assess the impact of the sacrificial phase on the final morphology and evaluate the ability of Cu atoms to re-arrange even at low Cu loading.

### 2.3. Preferential De-Alloying Regions Distinguished by Microstructure Analysis

Finally, the impact of the grain boundaries on the DA process was investigated. This was performed by physically marking the alloy samples with a Ga FIB prior to performing EBSD mapping on their surface. The samples were subsequently de-alloyed outside the SEM prior to being re-examined and re-mapped on the exact same position to evaluate grain orientation changes, pitting and the stability and impact of the grain boundaries on the pore propagation. As seen from the Figure 8, the commercial CuZn_30_ thin films were very likely fabricated by rolling from casing bulk material. The texture of this alloy, was found to be very strong in (311) as opposed to that of previously reported research with a (100) preferential orientation [30,54]. The surface crystallography analysis on CuZn_30_ thin film revealed that the pristine CuZn_30_ metal thin films exhibited a large grain distribution from 0.8 μm to approximately 10 μm with an average grain size just below 1 μm. The distribution of grain orientation is relatively narrow due to the texture of the single phase alloy. A number of larger grainer, typically larger than 3 μm exhibiting similar grain orientation formed linear patterns grid across the surface and cross section of the alloy matrix while the spaces between these elongated grains were typically composed of more spherical and much smaller grains with varied orientation. It was previously reported that the grain orientation distribution across Mg-Cd alloys may have a significant impact on the pore formation process [30] due to variations of d-spacing and lattice distances depending on the surface exposed crystalline facete [36].

**Figure 9:**
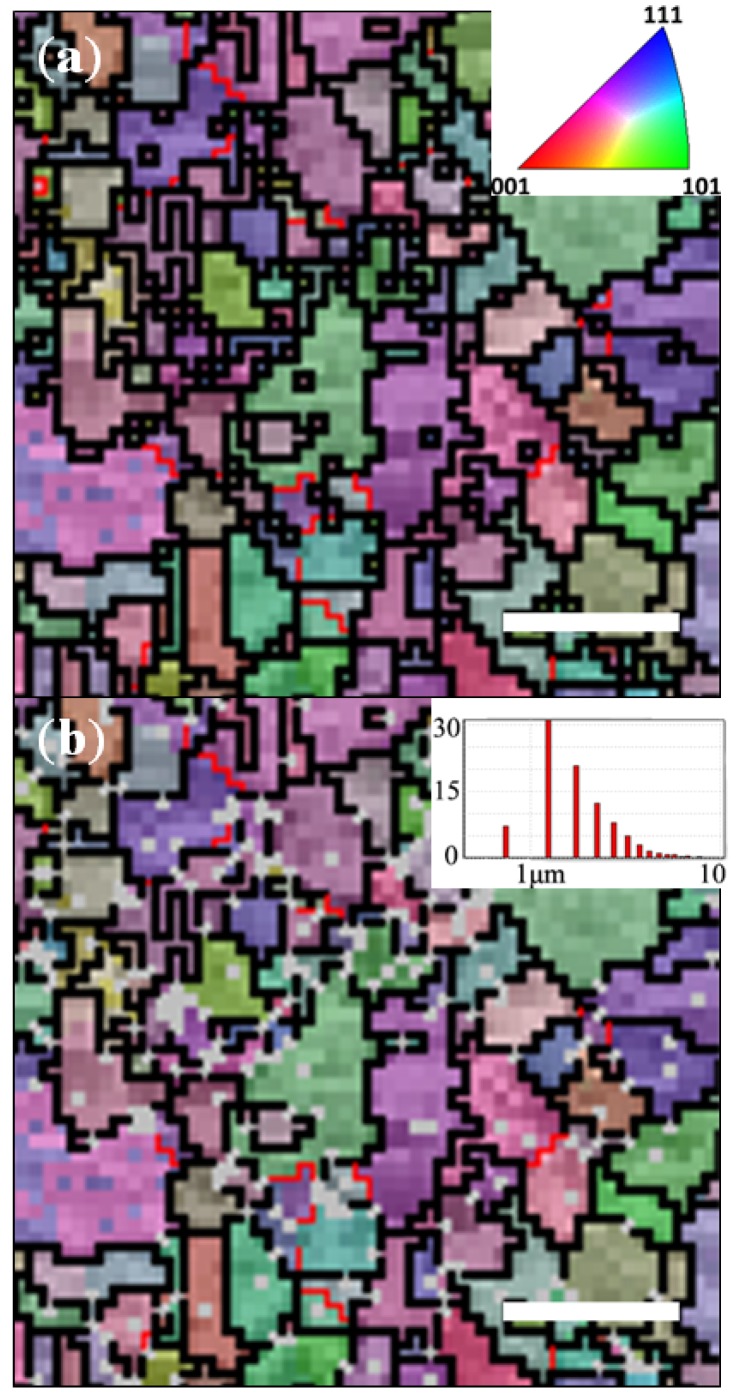
Electron back-scattered diffraction (EBSD) mapping on CuZn_30_ alloy: (**a**) before DA; and (**b**) after DA. Scale bars correspond to 5 μm. See Figure S10 for full scale image.

In-position DA of the CuZn_30_ metal thin films as seen in Figure 9a shows that the DA process was preferentially initiated across high density of small grains area. There is here no evidence that a specific orientation of grain was preferentially etched away to initiate pitting. As seen on Figure 9b, after DA, a number on unassigned pixels were marked in white. These unassigned EBSD spectra across the maps correspond to rough and non-planar areas where pitting was initiated. The larger angles of diffraction of the E-beam on these spots could not be integrated with the EBSD detector. These spots, examined at high electron microscopy (EM) magnification clearly correspond to early stages of surface pore formation. These spots, or typically referred to as nil pixels, are particularly concentrated on the grain boundaries and rarely occur across single grains. Although initially assumed as partially random, it appears that the generation of pits is privileged on less crystalline areas where more disordered atoms and larger inter-planar spacing are present. As a result, upon DA, the smaller grains across the surface of the CuZn_30_ thin films will be first etched from the surface and form dead end pits upon passivation of their surface. The size and depth of these grains is in fact in good agreement with the surface pores obtained in alkaline DA of these alloys. These boundary pits will therefore grow faster than to the ones formed across individual grains, grow around the smaller grains and ultimately leading to their detachment from the surface. This is an important result since it suggests that these pits, precursors of pores, will grow favorably where metal atoms exhibit the larger degree of disorder and not necessarily at the site of Zn rich grains.

**Figure 10:**
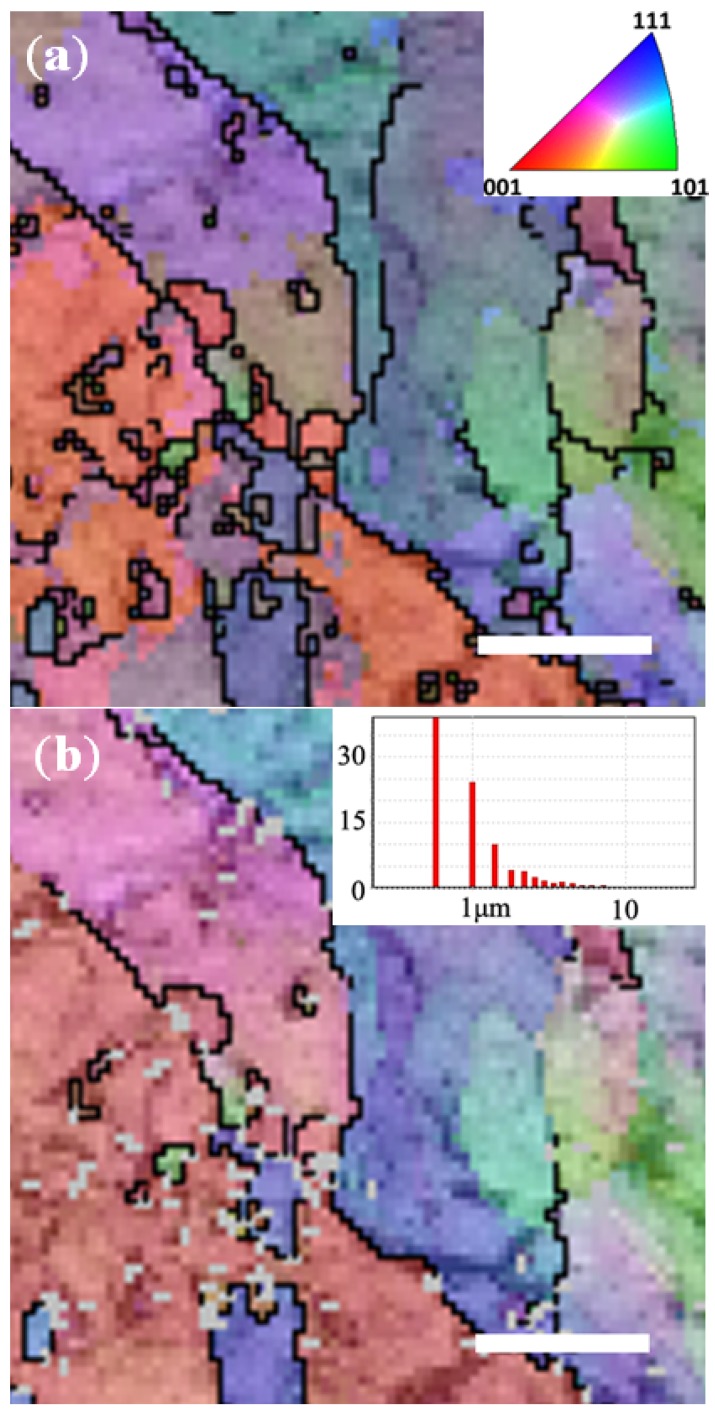
EBSD mapping of CuZn_15_: (**a)** before DA; and (**b**) after DA. Scale bars correspond to 5 μm. See Figure S11 for full scale image.

As opposed to the CuZn_30_ thin films, the CuZn_15_ ultra-thin foils were found to exhibit through pores similar to these obtained for the AuAg_50_ ultra-thin foils (Figure 4). This could be related to a number of reasons including the fact that the CuZn_15_ foils are up to 100 times thinner that the CuZn_30_ ones that the grains across the ultra-thin foil was found to be elliptically shaped leading to more stable ligament formation and much less CP across the material surface and pores. The CuZn_15_ ultra-thin foil microstructure is shown in Figure 10 to contain less grain boundaries but larger amounts of discontinuous sub-grain boundaries. In position DA of the CuZn_15_ ultra-thin foils shown in Figure 10 demonstrates again that the nil pixels appeared after DA on the grain boundaries and clearly visible sub-boundaries across the larger grains. Here most of the nil pixels appeared on the (100) oriented grains which are here axed parallel to the projection direction of the map. The more compact nature of the metal atoms across the (311) axis, and thus the d-spacing for etching agent to attack Zn metal, is larger than that of the (100) axis of the brass crystalline structure. The CuZn_15_ ultra-thin foils are primarily composed of (100) grains thus leading to a more stable surface to DA. The loose arrangement of atoms across the (311) may also lead to a higher energy surface more prone to etching and Cu atoms re-arrangement. Therefore, through pores could be more sustainably achieved across the CuZn_15_ ultra-thin foils due to both the foil micro-structure and the much thinner nature of the foils compared to the CuZn_30_ metal films.

Interestingly, specific sites for preferential DA have not been identified to date in the literature. This may be related to the type of fabrication process of previously studied alloys, leading to larger grain distributions and to a lower surface of volume ratio of grain boundaries. These preferential DA pitting sites shown in the present work may limit the possibility to form through pores across the CuZn_30_ thin films since they act as defect points and lead to complete grains removal until passivation due to too high CP across the liquid in the vicinity of the pore surface. Therefore, the performance of DA process perhaps will be improved by executing thermal pre-treatments, in which the preferential DA areas will be reduced by the coarsening of grains. This may reduce the grain boundary volume ratio across the material and lead to more homogeneous and stable porous structures with through pores. This strategy may open new routes for the formation of meso-porous metal materials through the fine control of the pristine alloy microstructure control.

## 3. Experimental Section

### 3.1. Pristine Alloy Materials and Chemicals

The pristine alloy thin films were CuZn_30_ (Cu 70 wt% and Zn 30 wt%, 25 μm thick) a is single phase commercial alpha-brass foil with highest zinc mass ratio and fabricated by rolling from casting bulk, and AuAg_50_ (Au 50 wt% and Ag 50 wt%, 120 nm thick) and CuZn_15_ (Cu 85 wt% and Zn 15 wt%, 150 nm thick) alloy leaves respectively purchased from Alfa-Aesar (Ward Hill, MA, USA) and Sepp Leaf Products Inc. (New York, NY, USA). The alloy leaves were processed by cold deformation and exhibit a (100) texture. All other chemicals used in this study including 32 wt% hydrochloric acid, 70% nitric acid, methanol and sodium hydroxide were of analytical grade and purchased from Sigma-Aldrich (St. Louis, MO, USA).

### 3.2. Surface Polishing

The raw CuZn_30_ foils were electro-polished with a 70 vol% methanol and 30 vol% nitric acid solution at 40 V for up to 60 s following a previously described procedure [62]. This polishing step was performed in order to smoothen the surface of the pristine foil which was found to be too rough for metal grain size distribution analysis.

### 3.3. Materials Characterization Techniques

Scanning electron micrographs (SEMs) were acquired on a Zeiss Supra 55VP Field-Emission Electron Microscope (Jena, Germany) with either a secondary electron detector at 5 keV for a 10 mm working distance or an In-lens detector at 5 keV and a 5 mm working distance for low or for high magnification imaging respectively. The DA samples were all thoroughly rinsed with DI water and ethanol and dried for at least 12 h at low vacuum prior to mounting on aluminum SEM stubs with carbon tape. The conductive samples were not coated but Ag paste was used to improve contact with the stub. The surface composition analysis was performed by EDS fitted on the SEM (Oxford X-Max 20 SDD, Oxfordshire, UK) and analysis performed with the Aztec Software (Oxfordshire, UK) at 20 keV in high current mode for a 10 mm working distance. Furthermore, the crystalline orientation analysis was performed with an Oxford (HKL) Nordlys S Electron Back-Scatter Diffraction (EBSD) Detector (Oxfordshire, UK) fitted on the Supra SEM with the Aztec Software. Tests were performed at 20 keV in high current mode, for a step size of 0.4 µm and exposure time determined by Aztec Software according to the quality of sample. Surface pore size distributions were analyzed from the acquired SEMs. However due to the irregularity of the pore size distributions, an average equivalent pore distribution was obtained by assuming pore size is the diameter of equivalent circle (Figures S12–S14 and Equation (S1)–(S3)).

Cross sections and surface patterning of the DA samples was performed with a FIB SEM (FEI Quanta 3D FEG FIB SEM, Hillsboro, OR, USA) with a Ga ion source at 30 keV and a 10 mm working distance. The cross-section preparation consisted of three consecutive FIB milling steps with a first one at a current of 30 nA prior to two cleaning steps at 1 nA and 0.5 nA, respectively. These cleaning steps were required to polish the surface of the sample *in situ* and remove grooves formed during the initial milling step due to the high Ga beam current. Furthermore, in order to study the impact of the microstructure on the final DA sample morphology the pristine foil was marked with a cross (Figure S15) with the FIB of Ga on the FEI Quanta and the same area always analyzed with the Zeiss Supra SEM and the Oxford EBSD Detector. A piece of silicon wafer (5 × 5 mm^2^) was used as a smooth substrate under the ultra-thin foil in order to ensure atomically smooth surface prior to marking and EBSD spectra acquisition for the AuAg_50_ and CuZn_15_ alloy thin films. The thickness of the metal alloy thin films was measured from the cross-section SEMs and correlated to thickness measurements obtained from a PST SUM2-025 Digital Micrometer (Kirwan, Australia) in order to assess from the roughness of the material surface.

Crystallography analysis was performed by XRD on a Panalytical X’pert Pro (Sydney, Australia) with a 0.04° step size per second at 40 kV. The data were analyzed with X’Pert HighScore Plus Software (Sydney, Australia). Peaks refinements were performed on the samples before and after DA to assess the formation of metal oxide surface layers as well as potential shifts across the crystalline phases due to the less noble metal atoms leaching from the matrix. The samples were mounted onto aluminum oxide sample holder. CuZn_30_ sample mounted with double side tape while AuAg_50_ and CuZn_15_ foil were directly adsorbed by electrostatic to the sample holder. SAXS experiment was performed in Australian Synchrotron (AS, Melbourne, Australia) on the SAXS/WAXS Beam Line. The scattering patterns were analyzed with Scatterbrain 2.1 (Melbourne, Australia) supplied from the SAXS technical group at the AS following procedures previously described [63]. The detector was a Pilatus 1M (Melbourne, Australia), the camera length was 1000 mm for a beam energy of 16 keV.

High magnification in small area (25 μm^2^) was performed by Bruker Multimode 8 Atomic Force Microscopy (AFM) at taping mode with tips (RTESPA, MPP-11120-10) from Bruker Co. (Billerica, MA, USA) which scan rate was 0.5 Hz. Low magnification in large area (1 mm^2^) was performed by AltiSurf 500 with CCS Prima Sensor and CL2 Probe which was performed by Altimet SAS (Thonon-Les-Bains, France).

Thermal conductivity of the de-alloyed samples was measured by linear conduction with a Gunt WL372 Heat Conduction Unit (Barsbüttel, Germany). The samples were processed as 25 mm disks. A reference sample was used to evaluate the variation of thermal conductivity through a procedure previously described [64]. The heating power was set as 40 W for a continuous water cooling flow of 1 L·h^−1^. Before collecting the conductivity data, the temperature of the feed side was stabilized for 15 min.

Brunner-Emmet-Teller (BET) surface area analysis was performed by Tristar 3000 with N_2_ (North Gosford, Australia). The de-alloyed samples were collected from etching solution and washed by DI water for three times, then move to BET tubes. Pristine sample and de-alloyed samples were left in oven at 80 °C though night for drying. Before test, all samples have degased at 150 °C for 60 min. A twenty-pressure point BET programs were used for BET surface area tests.

### 3.4. Chemical De-Alloying Process

#### 3.4.1. Dipping De-Alloying Tests

First, the chemical DA process was performed by dipping the pristine alloy thin foils into an etching solution for a fixed duration. The pH of the etching solutions was fixed by adjusting the concentration of either hydrochloric acid [60] or sodium hydroxide [40] at concentrations comprised between 0.01 mol·L^−1^ to 10 mol·L^−1^. The volume of the etching solution was fixed at 200 mL to limit the concentration and activity of the solubilized metal ions in solution. DA tests were performed at temperatures ranging between 298 K and 353 K to assess the impact of temperature on the final material morphology, the solubilization and atomic rearrangement processes. A water bath and reflux systems were used to heat up the solution and condense the evaporating water to maintain concentrations constant. All samples were de-alloyed under stirring with a magnetic stir bar at a rate of 80 rpm to ensure homogeneity of the solution. Since pH values of 1 M HCl and 1 M NaOH was 0 and 14, respectively, the pH values of 10 M HCl and 10 M NaOH were respectively set to −1 and 15 in order to simplify the explanation.

#### 3.4.2. Cross Flow Solution De-Alloying Tests

A cross-flow rig (Figure 7) was designed to evaluate the impact of metal ions CP on the surface and across the pores during the DA process. The etching solution was pumped through the module containing the sample with a peristaltic pump (Thermo Scientific FH100M Multichannel Peristaltic Pumps, Waltham, MA, USA) at a rate of 20 mL·min^−1^ or 72 mL·min^−1^. Two different configurations were tested to evaluate the impact of Zn and Cu ions on DA process, including: (i) a recirculating system where the etching solution was continuously recycled for the duration of the DA test; and (ii) a non-recirculating system where the etching solution exiting the module was directly disposed and not recycled. The later configuration offered the advantage over the former to offer a constant etchant concentration and a metal ions concentration close to nil thus maintain the activity of the solution constant over time.

#### 3.4.3. In-Position DA for Microscopy Analysis

In-position DA observations were performed by marking a polished sample prior to performing DA. This was performed by patterning the surface of the sample by FIB milling with a 2 μm deep cross. FIB milling is a smooth etching route that did not induce mechanical stress nor deform the sample and was shown to not alter the microstructure of the metal grains. This technique allowed for the surface analysis of the same physical area at different stages of the DA process. The sample of in-position DA were mounted on the same SEM stub and DA was performed without removing the sample without removing it from its aluminum stub to also limit potentially induced mechanical stress from peeling the sample from the supporting electrically conductive carbon tape. The DA tests were performed by dropping a fixed 100 µL volume of the etching solution on the surface of the sample. The etching solution was washed after 5 min of exposure with ethanol for three times prior to air blow-drying with nitrogen to remove remaining solvent and water. They were conditioned in vacuum for up to 15 min before being inserted within the SEM chamber.

## 4. Conclusions

In this paper, the chemical DA of commercial CuZn and AuAg alloys were systematically studied as a function of the materials micro-structure and process conditions. During the DA process, the pore size distribution was found to be largely related to the pore penetration depth across the material and to reach a maximum at typically around 2.5 µm across the 25 µm thick CuZn_30_ metal thin films. The depth of penetration was found not to change with exposure time or flow velocity but to be altered with the type and concentration of etchant, and thus pH, and to be increased for higher reaction temperatures. A critical thickness was therefore defined for the CuZn_30_ alloy studied which was attributed to the formation of surface oxide and potential passivation layer on the surface of the pores. The reducing of ion CP across the surface of the samples through non-recirculating etchant flows was found to prevent metal oxide formation on the surface of the films and to also increase by up to 50% the maximum depth of the pores. It is also demonstrated that the DA process preferentially occurs at the site of loose metal grain arrangement, such as grain boundaries or sub-boundaries. Small grain size distributions were shown to lead to larger densities of boundary area and thus to non-selective etching at these interfaces causing premature grains removal upon DA.

In future work, the relationship between the morphology and the thermo-mechanical and electrical properties and DA morphology should be evaluated to determine practical applications of de-alloyed materials. Furthermore, thermal treatment and controlled deformation can be used to adjust the microstructure of pristine alloy foil in order to achieve the formation of hierarchical structures.

## Supplementary Materials

Supplementary materials can be accessed at: http://www.mdpi.com/2079-4991/4/4/856/s1.
