# Supplementary material for: Impact of the De-Alloying Kinetics and Alloy Microstructure on the Final Morphology of De-Alloyed Meso-Porous Metal Films
Source: Nanomaterials (Basel). 2014 Oct 17;4(4):856–78. doi: 10.3390/nano4040856 (PMC5308459; doi:10.3390/nano4040856)
Supplement: Supplementary file 1 [file nanomaterials-04-00856-s001.pdf]

Supporting Information

Figure S1. Combined simplified E_h-pH diagram of Cu-Zn [1]. S1: both Cu and Zn are soluble; S2: only Zn is soluble and Cu is soluble only if solution is oxidizing solution; S3: both Cu and Zn are soluble; and S4: only Zn is soluble. Adapted from Figure 2 from [2] and Figure 9 from [3].

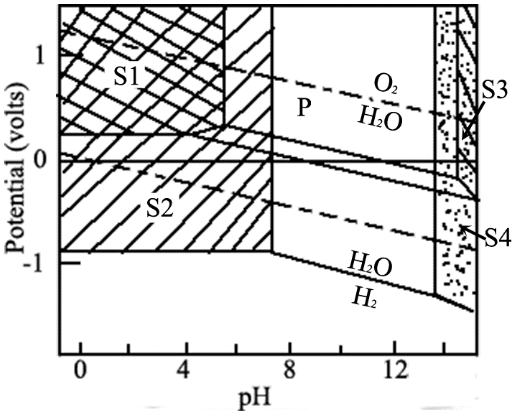

Figure S2. The scanning electron micrograph (SEM) images (in-plane view and cross-section view) of de-alloyed samples which de-alloyed with different etching solution revealing the grain microstructure and surface pitting. Scale bars for the surface and cross section views correspond to 500 nm and 10 μm, respectively.

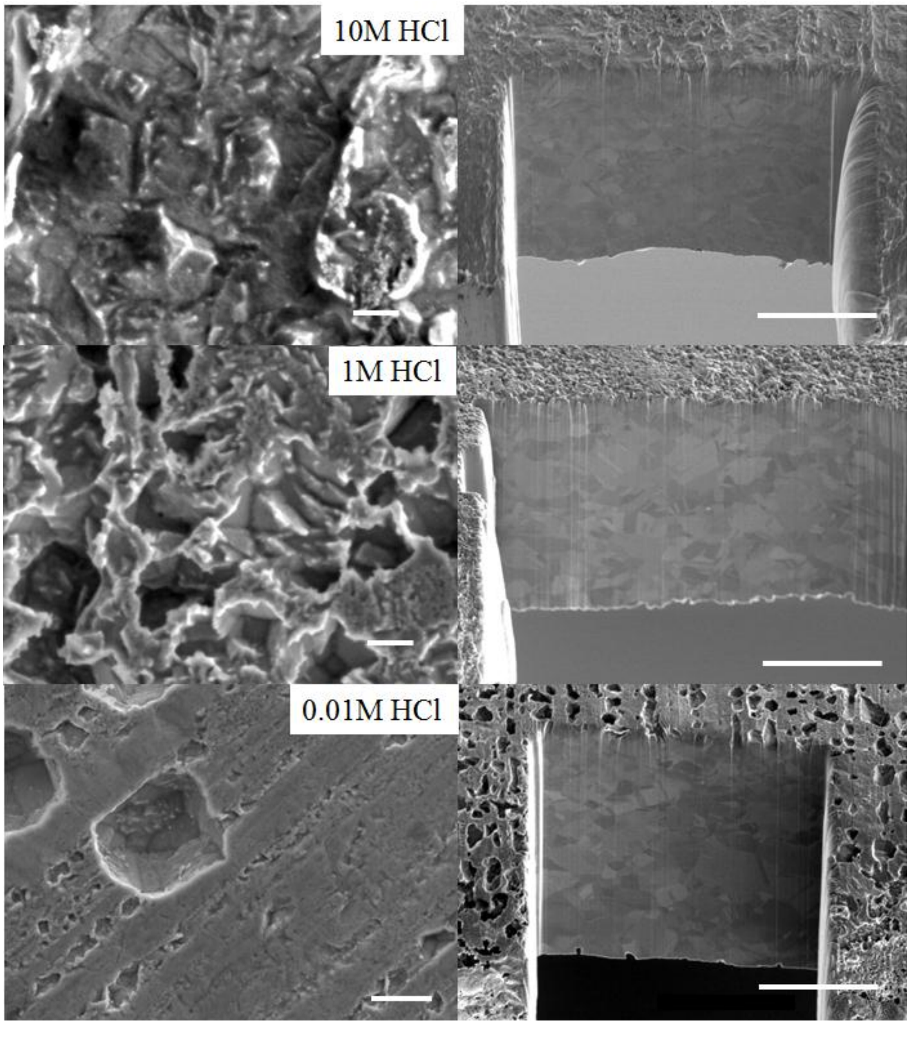

Figure S2. *Cont.*

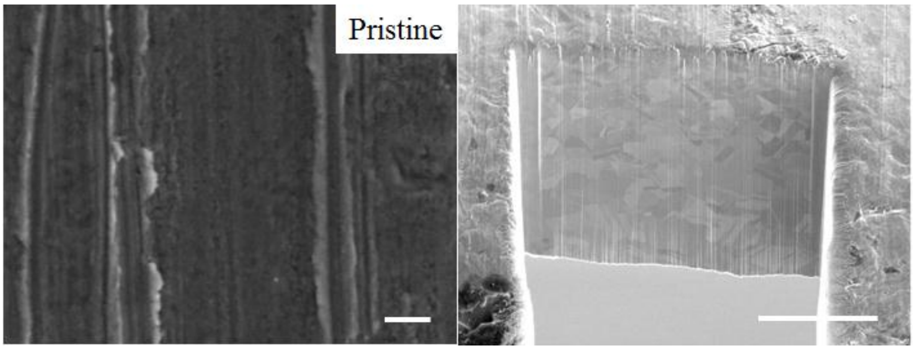

Figure S3. The SEMs (in-plane view and cross-section view) of de-alloyed samples which de-alloyed with different etching solutions and concentrations revealing the grain microstructure and surface pitting. Scale bars for the surface and cross section views correspond to 500 nm and 10 μm, respectively.

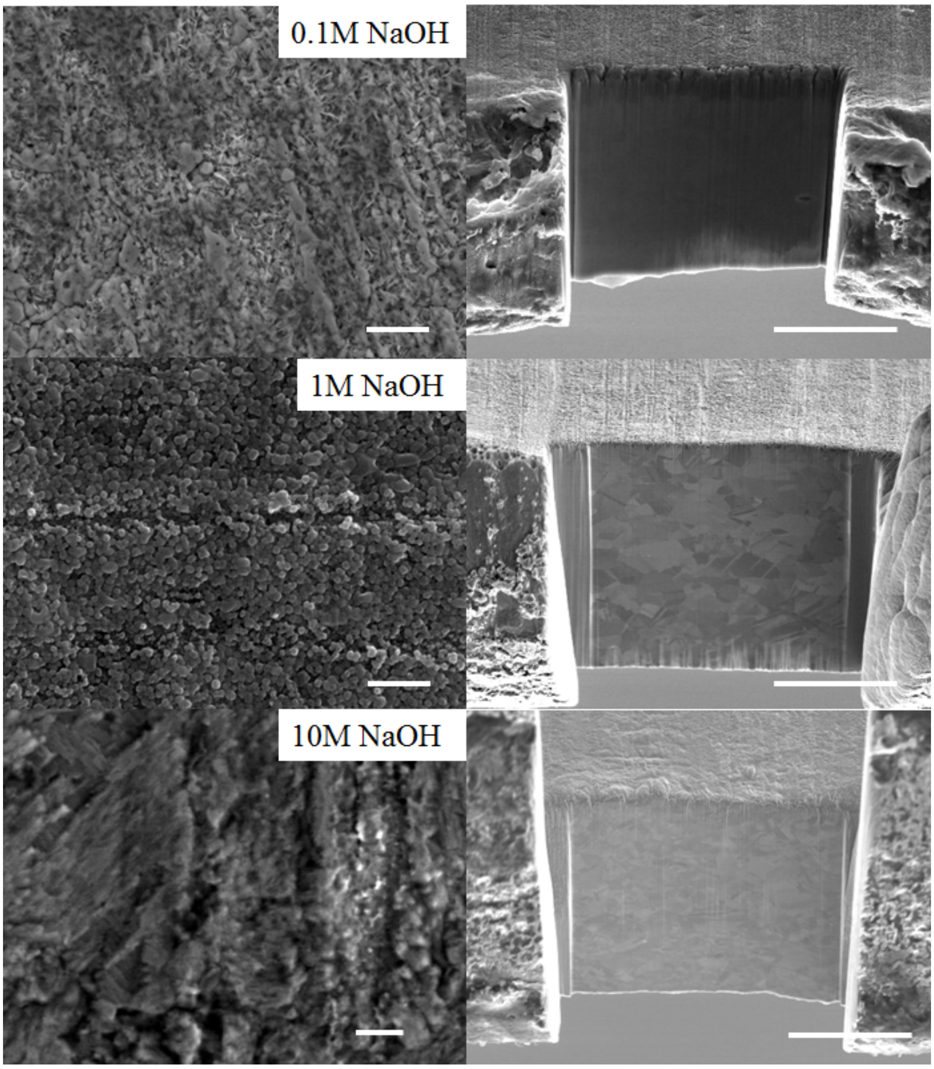

Figure S4. Energy dispersive spectroscopy (EDS) elemental distribution on the $CuZn_{30}$ de-alloying (DA) samples as a function of pH.

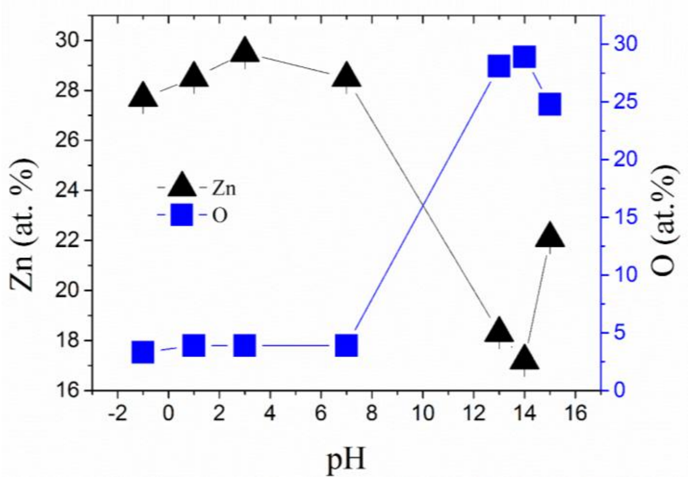

Figure S5. Small angle X-ray scattering (SAXS) patterns of *ex-situ* DA experiment of $CuZn_{15}$ showing precipitation on the samples surface.

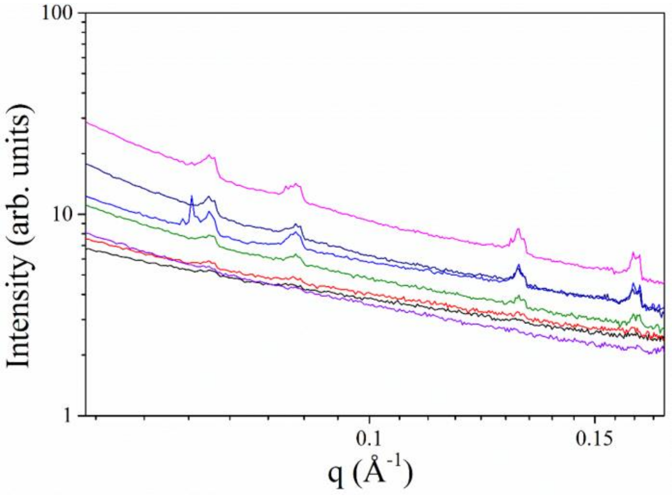

Figure S6. Relationship between the surface roughness, the thermal conductivity and the etching time for a 1 M NaOH solution at 25 ℃.

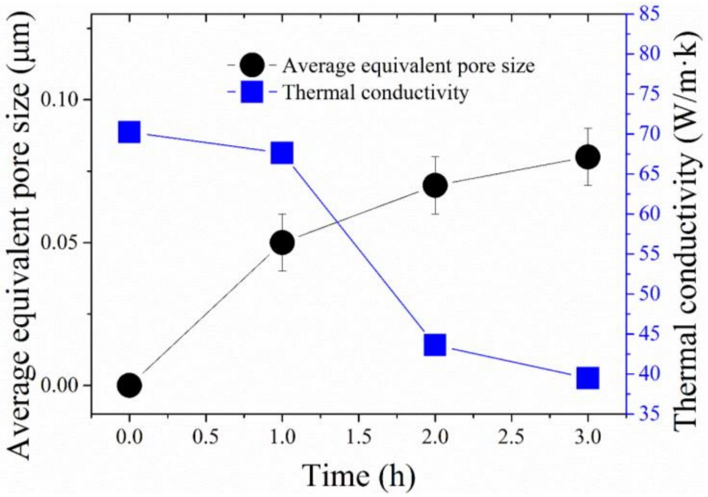

Figure S7. Surface topography evaluated by laser tomography (Altimet SAS AltiSurf 500 Model) and atomic force microscopy (AFM) mappings, with scanning performed before and after DA: (**a**) pristine surface with Altimet; (**b**) de-alloyed surface with Altimet; (**c**) Pristine surface by AFM; and (**d**) de-alloyed surface by AFM.

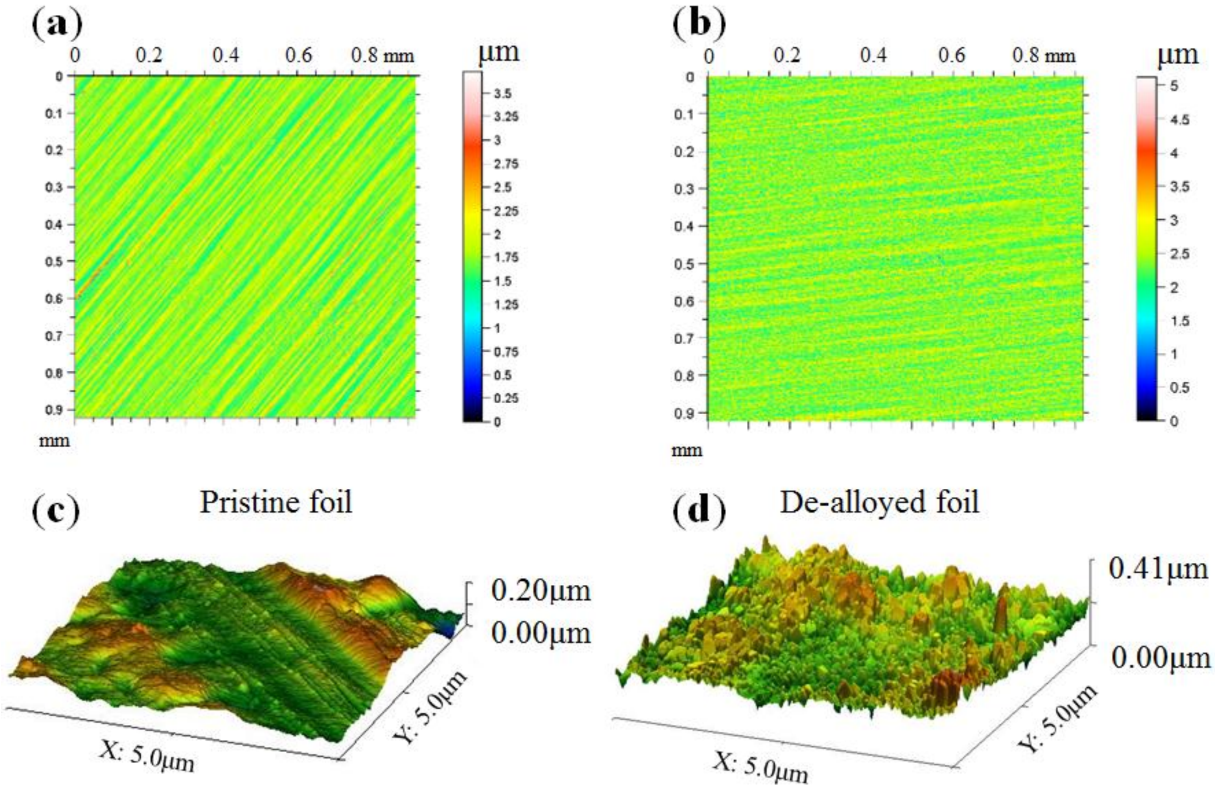

Figure S8. In-plane and cross-section views of a $CuZn_{30}$ samples de-alloyed with 1 M NaOH for 2.5 h at 25 ℃. Scale bars for the surface and cross section views correspond to 1 µm and 10 µm, respectively.

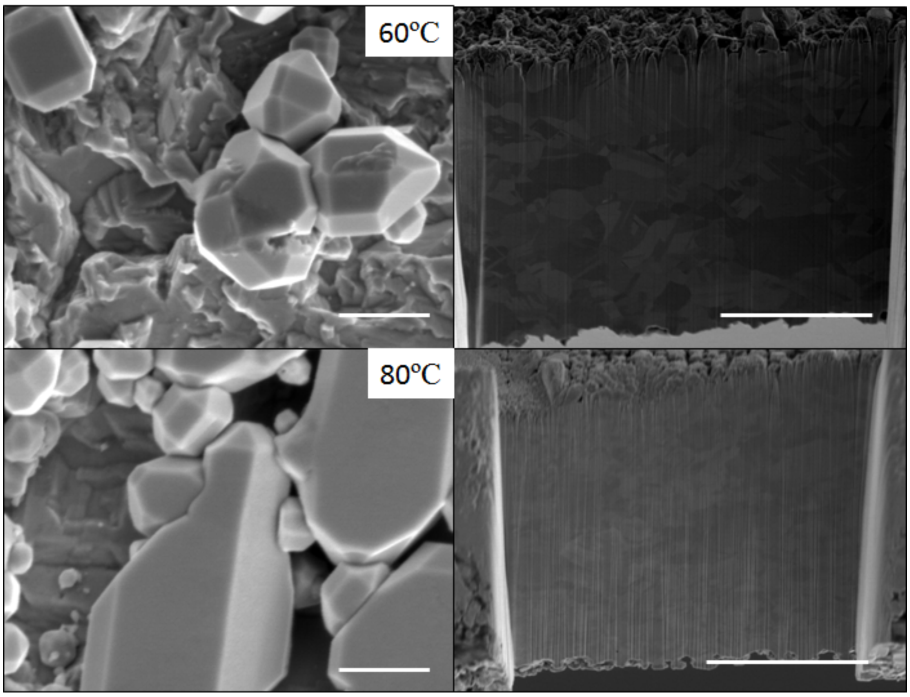

Figure S9. Schematic of through pore formation upon DA with: (**a**) pristine material; (**b**) pore initiation; (**c**) pore depth penetration increase prior to; (**d**) through pore formation; and (**e**) pore coarsening.

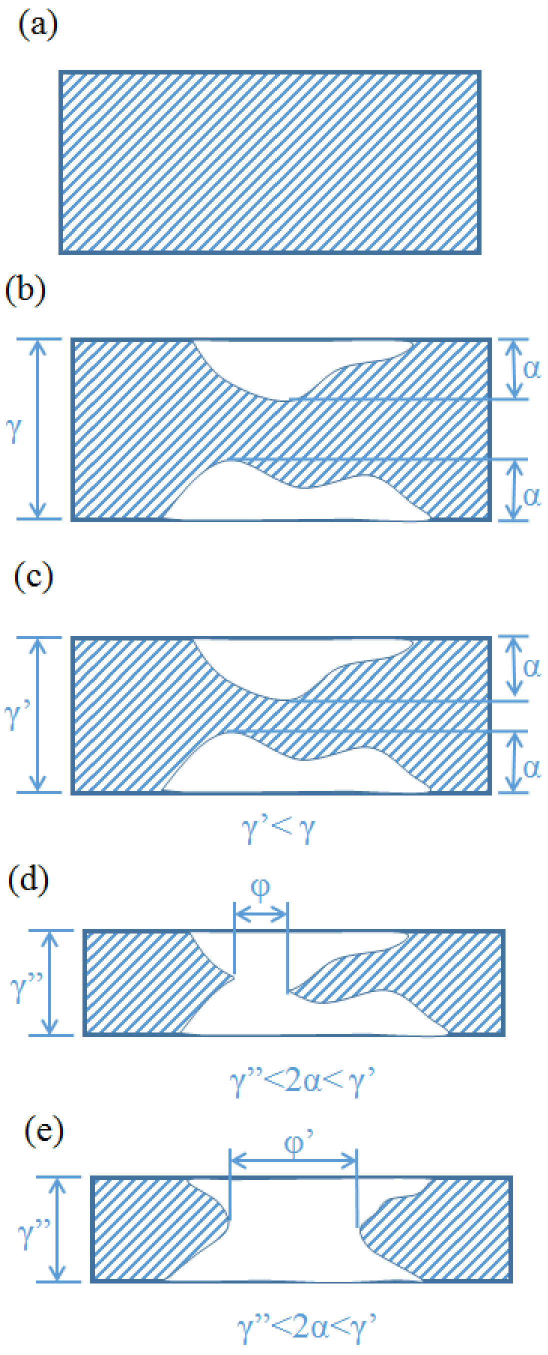

Figure S10. Electron back-scattered diffraction (EBSD) mapping on CuZn30 alloy: (**a**) before DA; and (**b**) after DA; and (**c**) grey-scale to highlight the preferential DA area. The texture colors are identified to different structure refinements. Non refined pixels correspond to rough parts of the samples, where DA occurred. The grain size distribution is shown for the sample in insert. Scale bars correspond to 20 µm.

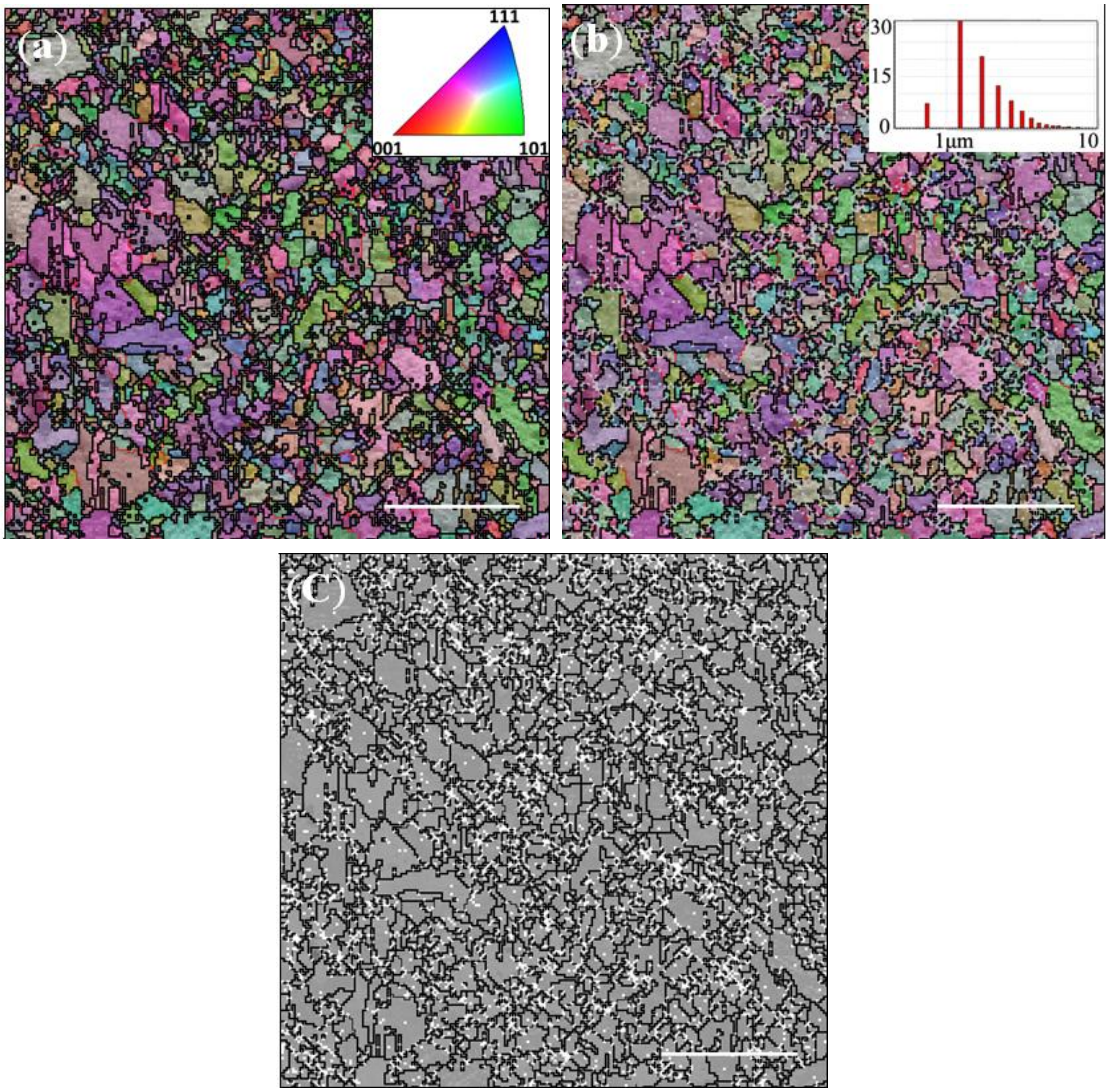

Figure S11. EBSD mapping of $CuZn_{15}$: (**a**) before DA; (**b**) after DA; and (**c**) grey-scale to highlight the preferential DA area. The texture colors are identified to different structure refinements. Non refined pixels correspond to rough parts of the samples, where DA occurred. The grain size distribution is shown for the sample in insert. Scale bars correspond to 20 µm.

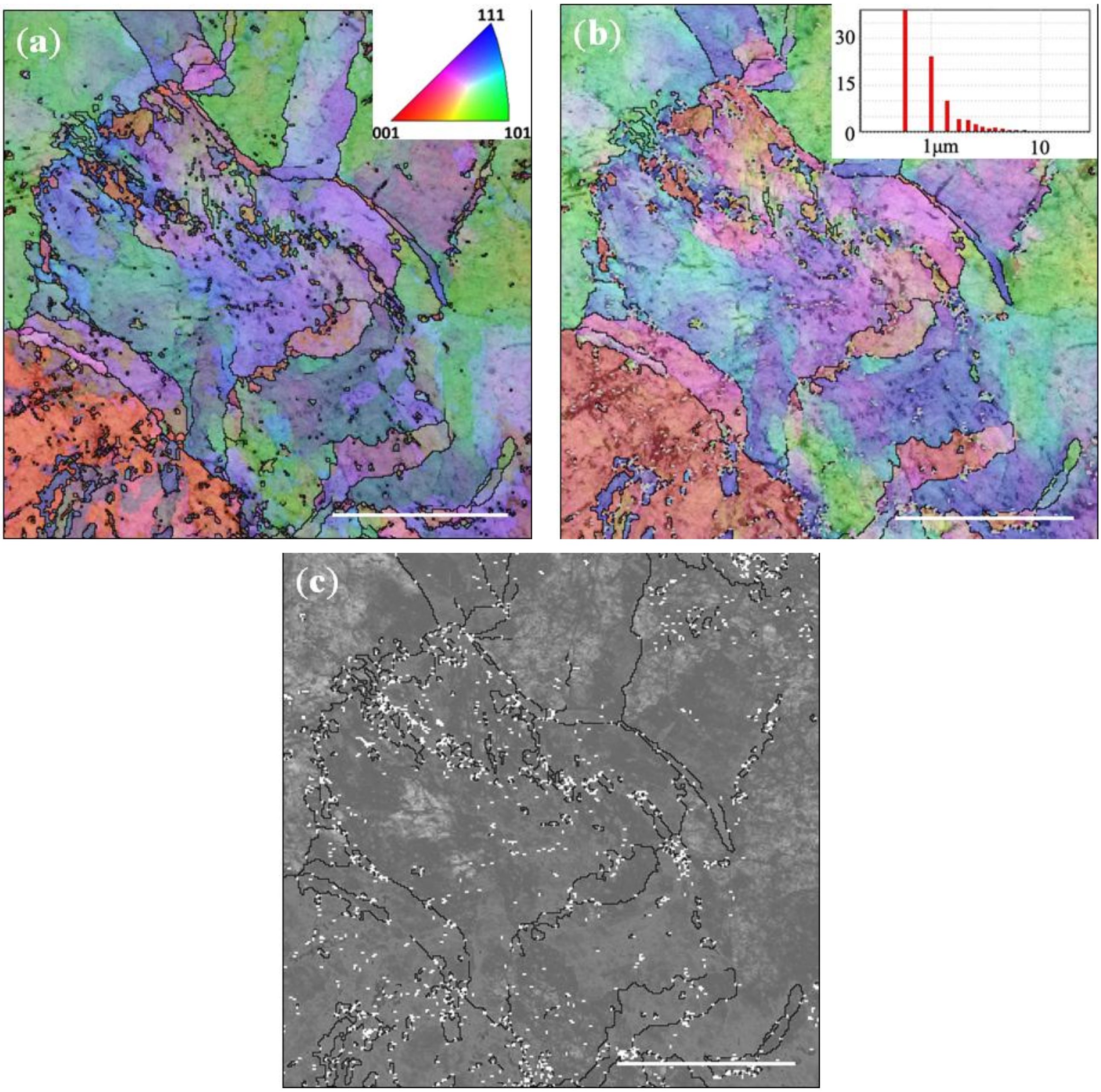

Pore Size Calculation from Scanning Electron Micrograph

Due to the irregularity of the pore size distributions, an average equivalent pore distribution was obtained by assuming pore size is the diameter of equivalent circle. The diameter of equivalent circle is equal to the approximate area of pore. The method of approximate are of pore has shown in following examples.

Figure S12. Square.

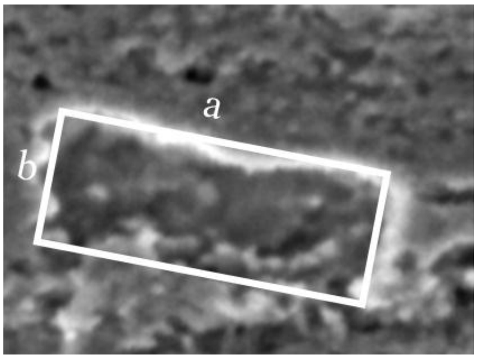

Formula:

$$d_e = \sqrt{\frac{4ab}{\pi}} \quad \text{(S1)}$$

where d_e is the equivalent diameter.

Figure S13. Triangle.

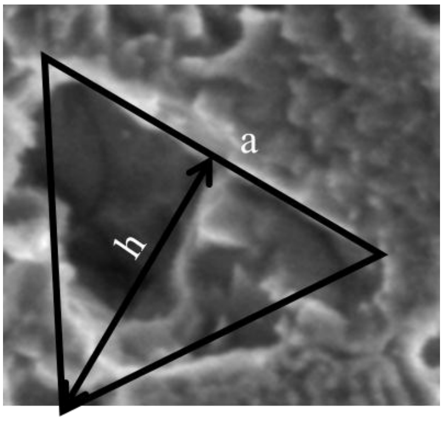

Formula:

$$d_e = \sqrt{\frac{2ah}{\pi}} \quad \text{(S2)}$$

where d_e is the equivalent diameter.

Figure S14. Long-shape.

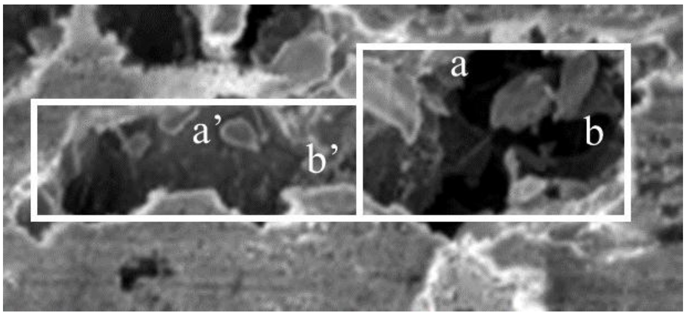

Formula:

$$d_e = \sqrt{\frac{4(a \times b + a' \times b')}{\pi}} \tag{S3}$$

where d_e is the equivalent diameter.

Figure S15. Patterning of the pristine sample by focus ion beam (FIB)—marking prior to EBSD. Scale bars correspond to 50 μm.

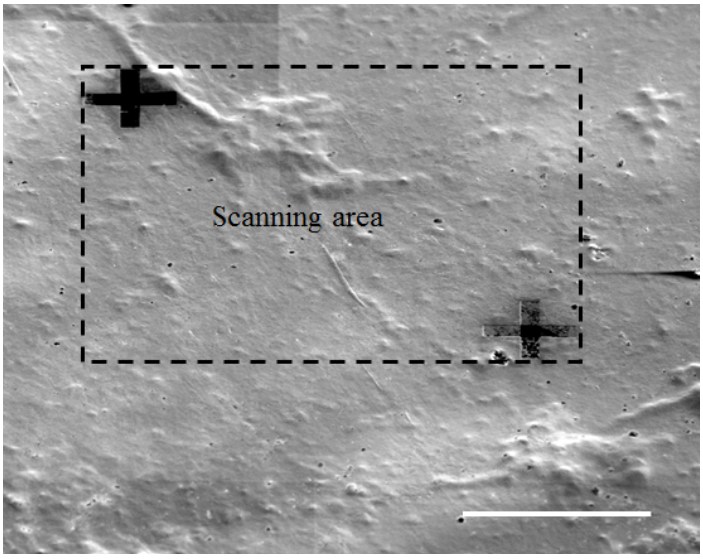

References

1. Huang, C.Y. *Corrosion and Protection of Metals*; Shanghai Jiao Tong University Press: Shanghai, China, 1989.
2. Al-Hinai, A.T.; Al-Hinai, M.H.; Dutta, J. Application of E_h-pH diagram for room temperature precipitation of zinc stannate microcubes in an aqueous media. *Mater. Res. Bull.* **2014**, *49*, 645–650.
3. Dong, C.S.; Gu, Y.; Zhong, M.-L.; Ma, M.-X.; Huang, T.; Liu, W.-J. Fabrication of nanoporous metal by selective electrochemical dealloying from laser cladding Cu-Mn alloys. *Acta Phys. Sin.* **2012**, *61*, doi:10.7498/aps.61.094211.

© 2014 by the authors; licensee MDPI, Basel, Switzerland. This article is an open access article distributed under the terms and conditions of the Creative Commons Attribution license (http://creativecommons.org/licenses/by/4.0/).
